# High multiple pregnancy rates after double embryo transfers in human: a retrospective cohort study

**DOI:** 10.1530/RAF-24-0078

**Published:** 2025-04-01

**Authors:** Jens Erik Dietrich, Ingrid Cáceres Valcárcel, Edison Capp, Thomas Strowitzki, Ariane Germeyer

**Affiliations:** ^1^ Heidelberg University Women´s Hospital, Department of Gynecologic Endocrinology and Fertility Disorders, Heidelberg, Germany; ^2^ Department of Obstetrics and Gynecology, Medicine School, Universidade Federal do Rio Grande do Sul, Porto Alegre, Brazil

**Keywords:** embryo transfer, multiple pregnancy rate, live birth rate, female age, embryo quality

## Abstract

**Abstract:**

To improve clinical outcomes of human medically assisted reproduction, the transfer of multiple embryos is frequently performed. This leads to high rates of multiple pregnancies. In this study we explored if a cohort of patients can be defined, that benefits from the transfer of two embryos while mitigating the odds of multiple pregnancies by considering female patients’ age, embryo quality and embryo cohort quality. In this retrospective cohort study, clinical pregnancy rate (CPR), live birth rate (LBR) and multiple pregnancy rate (MPR) after fresh single embryo transfers (SETs, *n* = 245) were compared to those after double embryo transfer (DET, *n* = 278). Female patient’s age, embryo quality and embryo cohort quality were used to explore clinical outcomes in subgroups. Overall, this study found that compared to SET, DET significantly increased the CPR (33.5 vs 49.6%, adjusted odds ratio (aOR): 2.233, 95% CI: 1.529–3.261, *P* < 0.001), LBR (24.1 vs 39.2%, aOR: 2.416, 1.605–3.636, *P* < 0.001) and MPR (0.0 vs 25.4%, *P* < 0.001). Subgroup analysis based on female age, embryo quality and further stratification based on embryo cohort score revealed that the MPR in all subgroups was high after DET and a subgroup with significantly reduced MPR after DET could not be defined. In conclusion, DETs are associated with high MPRs that cannot be avoided by considering female patients’ age, embryo quality and embryo cohort quality. SET is the most effective way to avoid a multiple pregnancy.

**Lay summary:**

This study examined whether it is possible to avoid multiple pregnancies in treatments of assisted reproduction when two embryos are transferred to certain patients. Transferring more than one embryo can increase the chances of pregnancy and live birth but also raises the risk of multiple pregnancies. Multiple pregnancies are linked to health risks for both the mother and the children. Using data from a single clinic, this study explored whether there are specific patients who could benefit from the transfer of two embryos while still having a lower risk of multiple pregnancy. However, the researchers found that DETs consistently lead to high rates of multiple pregnancies, even when factors like the mother’s age, embryo quality and overall embryo cohort quality are considered. The researchers concluded that transferring a single embryo remains the safest approach to prevent multiple pregnancies.

## Introduction

To improve clinical outcomes of medically assisted reproduction (MAR), the transfer of multiple embryos is frequently performed (Gliozheni *et al.* 2023). This leads to a significantly higher rate of multiple pregnancies after MAR, along with the associated increase of maternal, fetal and neonatal complications compared to singleton pregnancies (Eapen *et al.* 2020, Gliozheni *et al.* 2023, ESHRE Guideline Group on the Number of Embryos to Transfer *et al.* 2024). Consequently, several international and national professional bodies have recommended (elective) single embryo transfers (Practice Committee of the American Society for Reproductive Medicine and the Practice Committee for the Society for Assisted Reproductive Technologies 2021, ESHRE Guideline Group on the Number of Embryos to Transfer *et al.* 2024). In Europe, this resulted in a constant increase in the rate of single embryo transfers (SET) and a decrease in the rate of multiple pregnancies (Gliozheni *et al.* 2023). Among European countries reporting to the European IVF Monitoring Consortium (EIM), the overall rate of SET performed in 2019 was 55.4%, with large differences among the reporting countries, ranging from 100% to as low as 1.5% (Gliozheni *et al.* 2023). In Germany, the percentage of SET was 34.4%, double embryo transfer (DET) was 62.6% and triple embryo transfer was 3.0% (Gliozheni *et al.* 2023). This resulted in a twin rate of 18.1% and triplet rate of 0.4% (Gliozheni *et al.* 2023). Due to a further increase in the proportion of SET until 2021, the twin and triplet rates were further reduced to 15.2 and 0.3%, respectively (Barnitzky *et al.* 2024).

The continuing transfer of multiple embryos suggests that this strategy is still considered beneficial to improve the effectiveness in many MAR treatments, while accepting the safety concerns. Female patients’ age is often used as a predictor for clinical outcomes. Recommendations on the number of embryos to transfer by the ASRM are based on female patient age and modifiable by an individual prognosis (Practice Committee of the American Society for Reproductive Medicine and the Practice Committee for the Society for Assisted Reproductive Technologies 2021).

In addition to female patients’ age, embryo quality impacts clinical outcomes (Gardner *et al.* 2000, Racowsky *et al.* 2011, Vernon *et al.* 2011). Embryo morphology and kinetics can be assessed to predict the developmental potential before transfer (Ciray *et al.* 2014). Extended *in vitro* culture has been developed to identify the embryo with the highest developmental potential for elected single embryo transfers (eSETs) with the aim to improve clinical outcomes, while avoiding multiple pregnancies (Gardner & Schoolcraft 1999). This specific benefit is often ignored, leading to significantly higher rates of multiple pregnancies due to the transfer of more than one blastocyst (Cutting 2018).

In Germany, the Embryo Protection Act limits the number of embryos to be transferred up to three (Act for the Protection of Embryos 1990). In addition, the number of fertilized oocytes per cycle that can be cultured *in vitro* to embryos is limited and depends on the number of fertilized oocytes that is deemed necessary to obtain the desired number of viable embryos on the day of transfer (Taupitz & Hermes 2015, Nationale Akademie der Wissenschaften Leopoldina and Union der deutschen Akademien der Wissenschaften 2019). A typical eSET, in which all fertilized oocytes are cultured *in vitro*, irrespective of the total number, and a single embryo is selected from this group, is therefore not allowed in Germany (Nationale Akademie der Wissenschaften Leopoldina and Union der deutschen Akademien der Wissenschaften 2019). Furthermore, the number of embryos to be transferred is determined before the *in vitro* culture of embryos and takes predictive factors such as female patients’ age, medical conditions of the patient, prior pregnancies, the experience from previous MAR attempts and the personal wish of the patient into account.

Thus, in clinical practice, the number of embryos to be transferred is still mainly a decision based on patients’ and embryo characteristics to balance between effectiveness and safety of the treatment (Ma *et al.* 2022).

In this retrospective cohort study, we explored in fresh transfer cycles if a cohort of patients can be defined that benefits from DET in terms of clinical pregnancy and live birth rates (LBRs), while mitigating the odds of multiple pregnancy, based on clinical factors including female patients’ age, embryo quality and further stratification based on embryo cohort quality on the day of transfer after extended embryo culture.

## Materials and methods

### Study design

A retrospective cohort study was conducted at the Department of Gynecologic Endocrinology and Fertility Disorders, Heidelberg University Women’s Hospital, Ruprecht-Karl University Heidelberg, Germany. Between October 2016 and June 2020, all fresh cycle IVF- and ICSI-patients with embryos cultured up to day 4 or 5 and an embryo transfer with a known pregnancy outcome were retrospectively analyzed. Data were obtained from the prospectively maintained hospital electronic medical record system. Following the German Embryo Protection Act, the number of embryos cultured *in vitro* was limited. Up to five embryos were cultured, both for SET (mean: 3.3 ± 0.04, min. 1, max. 5) and for DET (mean: 4.0 ± 0.04, min. 2, max. 5). During the study period, *n* = 1,863 ovum pickups were performed. These led to *n* = 1,203 transfers, of which *n* = 754 were performed on day 4 or 5. Exclusion criteria were no embryo transfer (*n* = 660) or embryo transfer before day 4 (*n* = 449), female age >42 years on the day of transfer (*n* = 32), preimplantation genetic testing (*n* = 12), assisted hatching (*n* = 43) and calcium-ionophore treatment (*n* = 21). To reduce bias, only the first fresh cycle of each female patient within this period was included for the analysis, excluding additional transfers during the study period (*n* = 112). Additional cycles were excluded due to relevant co-medication (*n* = 2), missing data (*n* = 3), unknown pregnancy outcome (*n* = 5) and transfer of an additional thawed blastocyst (*n* = 1), leaving *n* = 523 transfers for the final analysis.

Outcomes were clinical pregnancy rate (CPR) (defined as the number of clinical pregnancies per number of embryo transfers), live birth rate (LBR) (defined as the number of live births per number of embryo transfers) and multiple pregnancy rate (MPR) (defined as the number of multiple pregnancies per number of clinical pregnancies).

### Controlled ovarian stimulation

The stimulation protocol was based on individual patients’ characteristics (e.g., ovarian reserve, polycystic ovary syndrome (PCOS) and endometriosis), time available and preference of the patient and physician (e.g. women with PCOS received mostly the gonadotropin-releasing hormone (GnRH) antagonist protocol, while women with endometriosis were more likely to receive the GnRH agonist protocol). A consistent rise in E2 levels and follicle growth was monitored until the presence of three or more follicles >17 mm in diameter. Ovum pick-up followed 36 h after ovulation induction by ultrasound-guided aspiration with a 17-gauge ovum aspiration needle (Cook, K-OSN-1730-B-90, Limerick, Ireland) and an aspiration pressure of 120 mmHg.

### Embryo grading

Embryo grading on day 4 and 5 was performed as previously described with modification (Roesner *et al.* 2017, Dietrich *et al.* 2020). Briefly, cleavage stage embryos were graded as A = stage-specific cell size and cytoplasmic fragmentation <10%; B = stage-specific cell size and cytoplasmic fragmentation 10–25%; C = cell size not stage-specific and/or cytoplasmic fragmentation 26–50%; and D = fragmentation >50%. Blastocysts were scored according to Gardner & Schoolcraft (1999). Good quality embryos (GQEs) were defined as 9–16-cell and grade A or B on day 4. Compacting or fully compacted morulae on day 4 were considered GQE if their quality was grade A or B on day 3 (independent of cell number on day 3). On day 5, GQE was defined as a blastocyst with an expansion 3–6, inner cell mass (ICM) quality A-B and trophectoderm (TE) quality A-B.

The embryo quality was translated into a score (embryo score) as follows. Embryo score 2 was attributed to GQEs, embryo score 1 was attributed to poor quality embryos (PQEs) that were considered for embryo transfer or cryopreservation and embryo score 0 was attributed to PQEs that were discarded. In the analyses, embryo score describes only the quality of the transferred embryos.

The embryo cohort quality is shown as a score calculated as the mean embryo score of all cultured embryos of the same treatment cycle. For analysis, the cohorts were grouped based on their embryo cohort score: 0.0–1.0, 1.1–1.5 and ≥1.6.

In addition to the assessment of morphology, time-lapse imaging was used to identify abnormally developing embryos not suitable for transfer. Conspicuous kinetics (such as reverse cleavage or direct cleavage) could lead to a deprioritization of embryos for transfer independent of morphological quality.

Embryos were transferred on day 4 or 5 to allow for more flexibility in scheduling the transfer. A previous study by our group showed that, in non-selected couples, a transfer on day 4 or 5 results in similar CPRs, ongoing pregnancy rates and MPRs (Holschbach *et al.* 2017).

### *In vitro*-culture

After IVF or ICSI embryos were cultured in Continuous Single Culture Complete (CSCM-C, 90165, FUJIFILM Irvine Scientific, FUJIFILM Europe B.V., The Netherlands) in an EmbryoScope (ES-D2, Vitrolife Sweden AB, Sweden) or EmbryoScope + (ES-P1, Vitrolife Sweden AB, Sweden) at 5% O_2_.

### Statistical analysis

Continuous variables were described by the mean values ± standard deviations (SD) and compared using independent samples *T*-test (two-sided p, equal variances not assumed). Categorical variables are presented with their absolute and relative numbers and associations were tested using Pearson chi-Square or Fisher’s exact test (exact sig., two-sided). Generalized linear model (binary logistic) with Wald chi-square statistics and a robust estimator for covariance was used to analyze binary clinical outcomes (clinical pregnancy, live birth or multiple pregnancies of DET). Covariates were analyzed by binary logistic regression with forward selection (likelihood ratio). Based on the results of this analysis, female age, embryo quality and female smoking habit were included in the model to calculate Exp(B), shown as adjusted odds ratio (aOR), and the lower and upper 95% Wald confidence interval for Exp(B) (95 CI). Subgroups with less than ten events were analyzed with a univariate model and results are shown as odds ratio (OR). *P*-values ≤0.05 were considered significant. The Institute of Medical Biometry (IMBI) of the Heidelberg University was consulted for advice on statistical analyses. Statistical analyses were performed using the SPSS Version 29.0 (SPSS, Inc, USA).

### Ethical approval

This study was approved by the Ethics Committee of the Medical Faculty Heidelberg (S-649/2016) and conducted according to the principles of the Declaration of Helsinki.

## Results

In our study population, the baseline characteristics female patients’ age, female smoking habit, endometrial thickness (<8 or ≥8 mm) and the number of cultured embryos were significantly different between SET and DET groups (Table 1). Body mass index (BMI), fertilization method, number of oocytes retrieved and number of fertilized oocytes (pronuclear stage oocytes, PNs) did not differ significantly between SET and DET groups (Table 1).

**Table 1:** Baseline characteristics of the study population. Continuous variables are shown as the mean ± SD (min – max) and were tested by independent samples *T*-test. Categorical variables are shown as number (percentage) and were tested by Pearson chi-Square (asymptotic significance, two-sided).

Variable	Total	SET	DET	*P*
*n*	523	245	*n* = 278	
Female age	35.0 ± 4.2 (24–42)	34.5 ± 4.6 (24–42)	35.5 ± 3.8 (24–42)	0.004
BMI (kg/m^2^)	23.7 ± 4.3 (16.0–38.0)	23.7 ± 4.2 (16.0–35.6)	23.7 ± 4.3 (16.3–38.0)	0.843
Female smoking				0.014
No	463 (88.5%)	208 (84.9%)	255 (91.7%)	
Yes	60 (11.5%)	37 (15.1%)	23 (8.3%)	
Number prior gravida	0.9 ± 1.4 (0–15)	0.9 ± 1.2 (0–6)	1.0 ± 1.5 (0–15)	0.556
Number prior para	0.3 ± 0.6 (0–4)	0.3 ± 0.6 (0–4)	0.3 ± 0.5 (0–3)	0.171
Number prior OPU	1.2 ± 1.8 (0–18)	0.8 ± 1.4 (0–7)	1.4 ± 2.1 (0–18)	<0.001
Number prior transfers	1.4 ± 2.4 (0–20)	1.0 ± 1.9 (0–12)	1.7 ± 2.7 (0–20)	<0.001
Fertilization method				0.694
IVF	235 (44.9%)	112 (45.7%)	123 (44.2%)	
ICSI	279 (53.3%)	130 (53.1%)	149 (53.6%)	
IVF/ICSI	9 (1.7%)	3 (1.2%)	6 (2.2%)	
Endometrial thickness				<0.001
<8 mm	44 (8.4%)	31 (12.7%)	13 (4.7%)	
≥8 mm	479 (91.6%)	214 (87.3%)	265 (95.3%)	
Number of oocytes	9.8 ± 4.7 (1–34)	10.0 ± 5.1 (1–34)	9.7 ± 4.4 (3–28)	0.397
Number of PNs	5.9 ± 2.9 (1–19)	5.9 ± 3.2 (1–19)	5.9 ± 2.5 (2–18)	0.896
Number cultured	3.7 ± 0.8 (1–5)	3.3 ± 0.7 (1–5)	4.0 ± 0.7 (2–5)	<0.001
Number of transfers on				0.932
D4	91 (17.4%)	43 (17.6%)	48 (17.3%)	
D5	432 (82.6%)	202 (82.4%)	230 (82.7%)	

BMI, body mass index; D4, day 4; D5, day 5; DET, double embryo transfer; Gravida, pregnancies; OPU, ovum pick-up; *P*, *P*-value; Para, live births; PNs, pronuclear stage oocytes; SET, single embryo transfer.

Binary logistic regression with forward selection (likelihood ratio) showed that clinical pregnancy and live birth were significantly affected by female age (OR: 0.931, 95% CI: 0.892–0.972, *P* = 0.001 and OR: 0.923, 95% CI: 0.882–0.966, *P* < 0.001), embryo quality (OR: 2.763, 95% CI: 1.733–4.406, *P* < 0.001 and OR: 3.248, 95% CI: 1.926–5.478, *P* < 0.001) and female smoking habit (OR: 0.504, 95% CI: 0.277–0.914, *P* = 0.024 and OR: 0.444, 95% CI: 0.227–0.864, *P* = 0.017). Clinical pregnancy or live birth were not significantly affected by female BMI (*P* = 0.292 or *P* = 0.439), endometrial thickness (*P* = 0.691 or *P* = 0.510), number of prior ovum pick-ups (*P* = 0.623 or *P* = 0.996), number of prior embryo transfers (*P* = 0.098 or *P* = 0.545), number of embryos cultured (*P* = 0.597 or *P* = 0.080), number of prior gravida (*P* = 0.407 or *P* = 0.677), number of prior para (*P* = 0.573 or *P* = 0.479) or the day of embryo transfer (*P* = 0.295 or *P* = 0.274), respectively.

After embryo transfer (*n* = 523), the CPR was 42.1% (*n* = 220/523) and the LBR was 32.1% (*n* = 168/523). The number of transferred embryos had a significant effect on clinical outcomes (Table 2A). The CPR was 33.5% (*n* = 82/245) after SET vs 49.6% (*n* = 138/278) after DET (aOR: 2.233, 95% CI: 1.529–3.261, *P* < 0.001). The LBR was 24.1% (*n* = 59/245) after SET vs 39.2% (*n* = 109/278) after DET (aOR: 2.416, 95% CI: 1.605–3.636, *P* < 0.001). The MPR was 0.0% (*n* = 0/82) after SET vs 25.4% (*n* = 35/138) after DET (*P* < 0.001).

**Table 2:** Clinical outcomes after SET or DET. Data are shown as ratio (percentage). CPR and LBR tested with a generalized linear model (binary logistic) including number and quality of embryos transferred, female age and smoking habit. MPR was tested with a Fisher’s exact test.

	CPR	aOR (95 CI)	*P*	LBR	aOR (95 CI)	*P*	MPR	*P* *
A: All patients								
SET	82/245 (33.5%)	1.00		59/245 (24.1%)	1.00		0/82 (0.0%)	
DET	138/278 (49.6%)	2.233 (1.529–3.261)	<0.001	109/278 (39.2%)	2.416 (1.605–3.636)	<0.001	35/138 (25.4%)	<0.001
B: Females <35 years								
SET	42/116 (36.2%)	1.0		33/116 (28.4%)	1.00		0/42 (0.0%)	
DET	65/111 (58.6%)	2.832 (1.589–5.047)	<0.001	54/111 (48.6%)	2.605 (1.448–4.686)	0.001	20/65 (30.8%)	<0.001
C: Females ≥35 years								
SET	40/129 (31.0%)	1.00		26/129 (20.2%)	1.00		0/40 (0.0%)	
DET	73/167 (43.7%)	1.716 (1.027–2.866)	0.039	55/167 (32.9%)	2.024 (1.143–3.585)	0.016	15/73 (20.5%)	<0.001

* Fisher’s exact test.

aOR (95 CI), adjusted odds ratio (95% confidence interval); CPR, clinical pregnancy rate; DET, double embryo transfer; LBR, live birth rate; MPR, multiple pregnancy rate; SET, single embryo transfer.

We then explored the dataset to test if a patients’ cohort can be identified that benefits significantly from DET while mitigating the odds of multiple pregnancy by performing subgroup analysis based on female age, embryo quality and cohort quality.

Considering female patients’ age, both younger (<35 years) and older (≥35 years) female patients had significantly higher CPR (36.2 vs 58.6%, aOR: 2.832, 95% CI: 1.589–5.047, *P* < 0.001 and 31.0 vs 43.7%, aOR: 1.716, 95% CI: 1.027–2.866, *P* = 0.039), LBR (28.4 vs 48.6%, aOR: 2.605, 95% CI: 1.448–4.686, *P* = 0.001 and 20.2 vs 32.9%, aOR: 2.024, 95% CI: 1.143–3.585, *P* = 0.016) and MPR (0.0 vs 30.8%, *P* < 0.001 and 0.0 vs 20.5%, *P* < 0.001) after DET compared to SET (SET vs DET) (Table 2B and C).

Further subgroup analysis considered embryo quality (Table 3). Compared to the transfer of a single PQE, the transfer of two PQEs did not significantly increase the CPR (18.9 vs 32.4%, OR: 2.057, 95% CI: 0.835–5.066, *P* = 0.117), but significantly increased the LBR (10.8 vs 27.0%, OR: 3.056, 95% CI: 1.089–8.575, *P* = 0.034) and MPR (0.0 vs 33.3%, *P* = 0.033) (Table 3A). Compared to the transfer of a single GQE, the transfer of an additional PQE did not significantly improve the CPR (39.8 vs 43.9%, OR: 1.184, 95% CI: 0.716–1.958, *P* = 0.510) or LBR (29.8 vs 28.6%, OR: 0.941, 95% CI: 0.545–1.627, *P* = 0.828), but the MPR was significantly increased (0.0 vs 23.3%, *P* < 0.001). Compared to the transfer of a single GQE, the transfer of two GQEs significantly increased the CPR (39.8 vs 58.0%, OR: 2.095, 95% CI: 1.334–3.292, *P* = 0.001), LBR (29.8 vs 49.7%, OR: 2.320, 95% CI: 1.460–3.688, *P* < 0.001) and MPR (0.0 vs 25.3%, *P* < 0.001) (Table 3A).

**Table 3:** Clinical outcomes after SET or DET based on embryo quality. Data are shown as ratio (percentage). CPR and LBR were tested with a generalized linear model (binary logistic). MPR was tested with a Fisher’s exact test.

	CPR	OR (95 CI)	*P*	LBR	OR (95 CI)	*P*	MPR	*P* *
A: All patients								
SET (1)	14/74 (18.9%)	1.00		8/74 (10.8%)	1.00		0/14 (0.0%)	
DET (1)	12/37 (32.4%)	2.057 (0.835–5.066)	0.117	10/37 (27.0%)	3.056 (1.089–8.575)	0.034	4/12 (33.3%)	0.033
SET (2)	68/171 (39.8%)	1.00		51/171 (29.8%)	1.00		0/68 (0.0%)	
DET (1.5)	43/98 (43.9%)	1.184 (0.716–1.958)	0.510	28/98 (28.6%)	0.941 (0.545–1.627)	0.828	10/43 (23.3%)	<0.001
DET (2)	83/143 (58.0%)	2.095 (1.334–3.292)	0.001	71/143 (49.7%)	2.320 (1.460–3.688)	<0.001	21/83 (25.3%)	<0.001
B: Females <35 years								
SET (1)	8/32 (25.0%)	1.00		6/32 (18.8%)	1.00		0/8 (0.0%)	
DET (1)	7/14 (50.0%)	3.000 (0.803–11.211)	0.102	6/14 (42.9%)	3.250 (0.816–12.937)	0.094	2/7 (28.6%)	0.200
SET (2)	34/84 (40.5%)	1.00		27/84 (32.1%)	1.00		0/34 (0.0%)	
DET (1.5)	18/37 (48.6%)	1.393 (0.640–3.033)	0.404	14/37 (37.8%)	1.285 (0.573–2.880)	0.542	6/18 (33.3%)	<0.001
DET (2)	40/60 (66.7%)	2.941 (1.473–5.872)	0.002	34/60 (56.7%)	2.761 (1.390–5.481)	0.004	12/40 (30.0%)	<0.001
C: Females ≥35 years								
SET (1)	6/42 (14.3%)	1.00		2/42 (4.8%)	1.00		0/6 (0.0%)	
DET (1)	5/23 (21.7%)	1.667 (0.448–6.207)	0.446	4/23 (17.4%)	4.211 (0.708–25.044)	0.114	2/5 (40.0%)	0.182
SET (2)	34/87 (39.1%)	1.00		24/87 (27.6%)	1.00		0/34 (0.0%)	
DET (1.5)	25/61 (41.0%)	1.083 (0.555–2.111)	0.816	14/61 (23.0%)	0.782 (0.366–1.671)	0.526	4/25 (16.0%)	0.028
DET (2)	43/83 (51.8%)	1.676 (0.911–3.081)	0.097	37/83 (44.6%)	2.111 (1.114–4.000)	0.022	9/43 (20.9%)	0.004

* Fisher’s exact test.

Embryo quality 1, poor quality embryo (PQE or PQE/PQE); 2, good quality embryo (GQE or GQE/GQE); 1.5, GQE/PQE; CPR, clinical pregnancy rate; DET, double embryo transfer; LBR, live birth rate; MPR, multiple pregnancy rate; OR (95 CI), odds ratio (95% confidence interval); SET, single embryo transfer.

Considering embryo quality and female age, there were similar trends for increased CPR, LBR and MPR in both younger (<35 years) and older (≥35 years) female patients after DET compared to SET (Table 3B and C).

To test if further stratification allows us to identify a cohort with significantly reduced MPR after DET, we considered the embryo cohort quality. Whereas embryo scores refer to the quality of transferred embryos only, the embryo cohort score is calculated as the mean quality score of all cultured embryos. Among cycles with a cohort score of 0–1.0 (*n* = 314), the scores of transferred embryos included SET (1 = PQE, *n* = 68), SET (2 = GQE, *n* = 89), DET (1 = PQE/PQE, *n* = 35), DET (1.5 = GQE/PQE, *n* = 88) and DET (2 = GQE/GQE, *n* = 34). Among cycles with a cohort score of 1.1–1.5 (*n* = 117), the embryo scores included SET (1, *n* = 4), SET (2, *n* = 40), DET (1, *n* = 2), DET (1.5, *n* = 7) and DET (2, *n* = 64). Among cycles with a cohort score of 1.6–2 (*n* = 92), the embryo scores included SET (1, *n* = 2), SET (2, *n* = 42), DET (1.5, *n* = 3) and DET (2, *n* = 45).

The CPR and LBR correlated to the cohort score, with higher scores resulting in higher CPR and LBR (Supplementary Table 1A, B, C (see section on Supplementary materials given at the end of the article)). After DET, the MPR was high in all cohort score subgroups; there was no subgroup with significantly reduced MPR (Table 4A, Supplementary Table 1A, B, C).

**Table 4:** Multiple pregnancy rates after the transfer of embryos depending on cohort score in all patients and in female patients <35 or ≥35 years. Data are shown as ratio (percentage) and were tested with a generalized linear model (binary logistic).

	All			<35 years			≥35 years		
	MPR	OR (95 CI)	*P*	MPR	OR (95 CI)	*P*	MPR	OR (95 CI)	*P*
A: Transfer of 2 embryos									
CS 0–1.0	14/66 (21.2%)	1.0		7/31 (22.6%)	1.0		7/35 (20.0%)	1.0	
CS 1.1–1.5	11/41 (26.8%)	1.362 (0.549–3.379)	0.505	6/18 (33.3%)	1.714 (0.471–6.240)	0.414	5/23 (21.7%)	1.111 (0.305–4.042)	0.873
CS 1.6–2	10/31 (32.3%)	1.769 (0.679–4.604)	0.243	7/16 (43.8%)	2.667 (0.728–9.764)	0.139	3/15 (20.0%)	1.000 (0.220–4.536)	1.000
B: Transfer of 2 GQE									
CS 0–1.0	2/16 (12.5%)	1.0		1/9 (11.1%)	1.0		1/7 (14.3%)	1.0	
CS 1.1–1.5	10/38 (26.3%)	2.500 0.481–12.994	0.276	5/16 (31.3%)	3.636 (0.353–37.457)	0.278	5/22 (22.7%)	1.765 (0.170–18.321)	0.634
CS 1.6–2	9/29 (31.0%)	3.150 (0.589–16.859)	0.180	6/15 (40.0%)	5.333 (0.523–54.344)	0.158	3/14 (21.4%)	1.636 (0.138–19.387)	0.696

CS, embryo cohort score; MPR, multiple pregnancy rate; OR (95 CI), odds ratio (95% confidence interval).

Stratification based on embryo quality and additionally based on embryo cohort quality was only performed for DET of GQE because case numbers of other combinations were too low for analysis. After the transfer of two GQEs, the CPR and LBR showed a trend corresponding to the cohort score, with higher cohort scores resulting in higher CPR and LBR (Supplementary Table 2A, B, C). After DET, the MPR was high in all cohort score subgroups; there was no subgroup with significantly reduced MPR (Table 4B, Supplementary Table 2A, B, C).

## Discussion

Data from international and national registries suggest that the transfer of multiple embryos is still performed in many treatment cycles in MAR (Barnitzky *et al.* 2024, Gliozheni *et al.* 2023). Our data confirm that overall, DET results in significantly higher CPR and LBR compared to SET. For the decision between SET and DET, the challenge is to find a balance between highest possible effectiveness and reducing the safety concern of multiple pregnancies with their associated maternal, fetal and neonatal risks.

Female patients’ age and embryo quality are known to affect the clinical outcome of MAR treatments (Gardner *et al.* 2000, Racowsky *et al.* 2011, Vernon *et al.* 2011, Vitagliano *et al.* 2023). Our data confirm that both female age and embryo quality significantly affect CPR and LBR.

Considering that in clinical practice embryos are usually transferred hierarchically with respect to embryo quality, we explored clinical outcomes for the transfer of a single PQE compared to DET of two PQEs or of a single GQE compared to DET of one GQE and one PQE or two GQEs. A trend for a better clinical outcome (CPR and LBR) was observed in most cases of DET, in line with results from a recent meta-analysis (Ma *et al.* 2022).

Our data show that PQE also have the potential to implant and give rise to live births, although at a reduced rate. Compared to SET (PQE), a DET (PQE/PQE) significantly improved the LBR, but compared to SET (GQE), a DET with an additional PQE did not significantly increase the CPR or LBR. These data support findings showing that the additional transfer of a PQE does not negatively affect clinical outcomes (Wintner *et al.* 2017, Dobson *et al.* 2018, Wang *et al.* 2020, Theodorou *et al.* 2021). However, in all cases, the additional transfer of a PQE increased the MPR, consistent with previous reports (Wintner *et al.* 2017, Dobson *et al.* 2018, Wang *et al.* 2020, Theodorou *et al.* 2021). Although there was no statistically significant difference in the MPR after DET (PQE/PQE) compared to SET (PQE) in the subgroup analysis based on female patients’ age, the MPR was high after DET. The lost significance in the subgroup analysis may be due to small numbers.

Thus, our data support the recommendation that the decision to perform DET should not be based on female patients’ age and embryo quality (ESHRE Guideline Group on the Number of Embryos to Transfer *et al.* 2024).

In this study, we explored if additional stratification could identify a cohort of patients with significantly reduced odds of multiple pregnancy. A previous study found that cycles with cryopreservation of supernumerary embryos resulted in increased LBRs compared to those without cryopreservation, suggesting that the availability of supernumerary embryos suitable for cryopreservation may indicate good quality (Stern *et al.* 2012). The quality of supernumerary embryos was shown to have an impact on the clinical outcome, where the availability of additional GQE led to a better clinical outcome (Salha *et al.* 2000). Machtinger *et al.* showed that the presence of a non-cleaved embryo was an indicator of an overall reduced quality of embryos in the cohort (Machtinger *et al.* 2015). In this study, we used the cohort score of all cultured embryos as a proxy for overall cycle quality and explored its association with the clinical outcome of MAR cycles. Although the cohort score is affected by the score of the transferred embryo (Romanski *et al.* 2018), especially in relatively small cohorts of *in vitro* cultured embryos, the cohort score does not only reflect the score of the transferred embryos. Supernumerary embryos can impact on the cohort score such that cycles with only GQE will have a higher cohort score than cycles where only the transferred embryos were GQE. Thus, we anticipated an additional useful stratification by considering the cohort score.

The clinical outcome after the transfer of GQE correlated to the cohort score. Transfer of GQE from cohorts with increasing scores had a trend for a better clinical outcome. An explanation for the improved outcome with additional stratification based on cohort score may be that cohort scores better reflect the quality of the embryos available for transfer than the simplified binary classification GQE vs PQE. GQE and PQE each include a variety of different embryo qualities. Cycles with a high cohort score have more embryos of good quality and with a hierarchical transfer strategy the best ones among GQE will be chosen for transfer. Thus, in agreement with findings by Romanski *et al.* we found that the cohort score may be useful to describe the overall quality of the cycle (Romanski *et al.* 2018). However, after DET, all subgroups had high MPR and stratification based on cohort score did not enable us to identify a subgroup with reduced odds of MPR. Thus, in this exploratory study, we were unable to define a subgroup based on female age, embryo quality or further stratification based on the embryo cohort quality with acceptable reduction of the MPR.

It is important to highlight that for multiple pregnancies, there was a quasi-complete separation of the data in our study population based on the number of embryos transferred (SET vs DET). Whereas no multiple pregnancies had occurred after SET, all subgroups with a DET were affected by multiple pregnancies. Therefore, the number of embryos transferred was the only variable that could reliably control the odds of a multiple pregnancy. This is expected, since after SET, twin pregnancies may occur only in the rare case of monozygotic twinning (Blickstein 2005). The spontaneous monozygotic twinning rate is estimated to being approximately 0.4% (Blickstein *et al.* 1999, Chen *et al.* 2023). In MAR, this rate is increased to 1.6% (Chen *et al.* 2023).

Considering effectiveness, an alternative strategy to the transfer of multiple embryos is sequential SET. With the improved outcome after cryopreservation, sequential treatment strategies with fresh and frozen-warmed cycles have become an option (Nagy *et al.* 2020). Considering cumulative pregnancy rates, SET and DET strategies result in a similar clinical outcome (Kamath *et al.* 2020). Thus, sequential SET appears to be as effective as DET, while mitigating the risks of a multiple pregnancy.

We acknowledge limitations in our study. A limiting factor is the restricted number of embryos that can be cultured due to legal regulations in Germany imposed by the German Embryo Protection Act (Act for the Protection of Embryos 1990, Taupitz & Hermes 2015, Nationale Akademie der Wissenschaften Leopoldina and Union der deutschen Akademien der Wissenschaften 2019). Because of that, the analyzed embryo cohort quality may not fully reflect the cycle quality. In this study, extended *in vitro* culture included transfers on day 4 and day 5, which may be a limitation. Female age was tested for groups <35 years and ≥35 years. Choosing different age categories may result in other outcomes. A further limitation is the retrospective and exploratory design including subgroup analyses and resulting in small numbers analyzed. Results require confirmation with larger numbers. This is a single center study, and outcomes may differ in other settings.

## Conclusion

Compared to single embryo transfer, the transfer of two embryos is associated with significantly increased CPRs and LBRs. The additional transfer of a PQE in DET did not negatively affect the clinical outcomes (CPR and LBR). Subgroup analysis based on female patients’ age, embryo quality and further stratification based on embryo cohort quality revealed that after DET, the MPR remained high in all subgroups. Considering the risks associated with multiple pregnancies, these data support the preference of SET over DET.

## Supplementary materials



## Declaration of interest

The authors declare that there is no conflict of interest that could be perceived as prejudicing the impartiality of the work reported.

## Funding

For the publication fee, we acknowledge financial support by Heidelberg University. E Capp is a recipient of a scholarship from CNPq–Conselho Nacional de Desenvolvimento Científico e Tecnológico, Brazil. No further funding was received for this study.

## Author contribution statement

JED helped in study conception and design, data acquisition, data analysis and interpretation, manuscript drafting and revision. ICV helped in study conception and design, data acquisition, data analysis and interpretation, manuscript drafting and revision. EC helped in data analysis and interpretation, manuscript drafting and revision. TS helped in study conception and design, data analysis and interpretation, manuscript revision. AG helped in study conception and design, data analysis and interpretation, manuscript drafting and revision. All authors gave final approval of the version to be published and agreement to be accountable for all aspects of the work in ensuring that questions related to the accuracy or integrity of any part of the work are appropriately investigated and resolved.
