# Supplementary material for: High multiple pregnancy rates after double embryo transfers in human: a retrospective cohort study
Source: Reprod Fertil. 2025 Apr 1;6(2):e240078. doi: 10.1530/RAF-24-0078 (PMC12002859; doi:10.1530/RAF-24-0078)
Supplement: Supplementary file 1 [file supplementary_materials.pdf]

Supplementary Table 1A: Clinical outcomes based on cohort score and number of embryos transferred.

SET	CPR	OR (95CI)	P	LBR	OR (95CI)	P	MPR	OR (95CI)	P
CS 0-1.0	44/157 (28.0 %)	1.0		28/157 (17.8 %)	1.0		0/44 (0.0 %)		
CS 1.1-1.5	18/44 (40.9 %)	1.778 (0.888-3.561)	0.104	15/44 (34.1 %)	2.383 (1.131-5.021)	0.022	0/18 (0.0 %)		
CS 1.6-2	20/44 (45.4 %)	2.140 (1.076-4.259)	0.030	16/44 (36.4 %)	2.633 (1.259-5.505)	0.010	0/20 (0.0 %)		
DET									
CS 0-1.0	66/157 (42.0 %)	1.0		47/157 (29.9 %)	1.0		14/66 (21.2 %)	1.0	
CS 1.1-1.5	41/73 (56.2 %)	1.767 (1.009-3.094)	0.047	34/73 (46.6 %)	2.040 (1.151-3.618)	0.015	11/41 (26.8 %)	1.362 (0.549-3.379)	0.505
CS 1.6-2	31/48 (64.6 %)	2.514 (1.285-4.919)	0.007	28/48 (58.3 %)	3.277 (1.680-6.389)	<0.001	10/31 (32.3 %)	1.769 (0.679-4.604)	0.243

Supplementary Table 1B: Clinical outcomes based on cohort score and number of embryos transferred in female patients < 35.

SET	CPR	OR (95CI)	P	LBR	OR (95CI)	P	MPR	OR (95CI)	P
CS 0-1.0	22/71 (31.0 %)	1.0		16/71 (22.5 %)	1.0		0/22 (0.0 %)		
CS 1.1-1.5	8/19 (42.1 %)	1.620 (0.572-4.585)	0.364	7/19 (36.8 %)	2.005 (0.677-5.939)	0.209	0/8 (0.0 %)		
CS 1.6-2	12/26 (46.2 %)	1.909 (0.760-4.793)	0.169	10/26 (38.5 %)	2.148 (0.817-5.648)	0.121	0/12 (0.0 %)		
DET									
CS 0-1.0	31/61 (50.8 %)	1.0		25/61 (41.0 %)	1.0		7/31 (22.6 %)	1.0	
CS 1.1-1.5	18/29 (62.1 %)	1.584 (0.642-3.905)	0.318	15/29 (51.7 %)	1.543 (0.634-3.754)	0.339	6/18 (33.3 %)	1.714 (0.471-6.240)	0.414
CS 1.6-2	16/21 (76.2 %)	3.097 (1.008-9.516)	0.048	14/21 (66.7 %)	2.880 (1.017-8.156)	0.046	7/16 (43.8 %)	2.667 (0.728-9.764)	0.139

Supplementary Table 1C: Clinical outcomes based on cohort score and number of embryos transferred in female patients ≥ 35.

SET	CPR	OR (95CI)	P	LBR	OR (95CI)	P	MPR	OR (95CI)	P
CS 0-1.0	22/86 (25.6 %)	1.0		12/86 (14.0 %)	1.0		0/22 (0.0 %)		
CS 1.1-1.5	10/25 (40.0 %)	1.939 (0.761-4.942)	0.165	8/25 (32.0 %)	2.902 (1.027-8.197)	0.044	0/10 (0.0 %)		
CS 1.6-2	8/18 (44.4 %)	2.327	0.114	6/18 (33.3 %)	3.083	0.056	0/8 (0.0 %)		

		(0.816-6.639)			(0.972-9.780)				
DET									
CS 0-1.0	35/96 (36.5 %)	1.0		22/96 (22.9 %)	1.0		7/35 (20.0 %)	1.0	
CS 1.1-1.5	23/44 (52.3 %)	1.909 (0.926-3.933)	0.080	19/44 (43.2 %)	2.556 (1.192-5.483)	0.016	5/23 (21.7 %)	1.111 (0.305-4.042)	0.873
CS 1.6-2	15/27 (55.6 %)	2.179 (0.917-5.176)	0.078	14/27 (51.9 %)	3.622 (1.484-8.842)	0.005	3/15 (20.0 %)	1.000 (0.220-4.536)	1.000

Data are shown as ratio (percentage) and were tested with a generalized linear model (binary logistic). CS = Embryo cohort score, CPR = clinical pregnancy rate, DET = double embryo, LBR = live birth rate, MPR = multiple pregnancy rate, OR (95CI) = odds ratio (95 % confidence interval), P = p-value, SET = single embryo transfer.

Supplementary Table 2A: Clinical outcomes based on cohort score and number of embryos transferred after the transfer of GQE.

SET (2)	CPR	OR (95CI)	P	LBR	OR (95CI)	P	MPR	OR (95CI)	P
CS 0-1.0	31/89 (34.8 %)	1.0		21/89 (23.6 %)	1.0		0/31 (0.0 %)		
CS 1.1-1.5	17/40 (42.5 %)	1.383 (0.644-2.968)	0.405	14/40 (35.0 %)	1.744 (0.773-3.933)	0.180	0/17 (0.0 %)		
CS 1.6-2	20/42 (47.6 %)	1.701 (0.806-3.587)	0.163	16/42 (38.1 %)	1.993 (0.903-4.399)	0.088	0/20 (0.0 %)		
DET (2)									
CS 0-1.0	16/34 (47.1 %)	1.0		13/34 (38.2 %)	1.0		2/16 (12.5 %)	1.0	
CS 1.1-1.5	38/64 (59.4 %)	1.644 (0.711-3.801)	0.245	32/64 (50.0 %)	1.615 (0.692-3.771)	0.267	10/38 (26.3 %)	2.500 (0.481-12.994)	0.276
CS 1.6-2	29/45 (64.4 %)	2.039 (0.822-5.060)	0.124	26/45 (57.8 %)	2.211 (0.890-5.492)	0.088	9/29 (31.0 %)	3.150 (0.589-16.859)	0.180

Supplementary Table 2B: Clinical outcomes based on cohort score and number of embryos transferred in female patients < 35 after the transfer of GQE.

SET (2)	CPR	OR (95CI)	P	LBR	OR (95CI)	P	MPR	OR (95CI)	P
CS 0-1.0	14/41 (34.1 %)	1.0		10/41 (24.4 %)	1.0		0/14 (0.0 %)		
CS 1.1-1.5	8/18 (44.4 %)	1.543 (0.497-4.785)	0.453	7/18 (38.9 %)	1.973 (0.603-6.457)	0.261	0/8 (0.0 %)		
CS 1.6-2	12/25 (48.0 %)	1.780 (0.645-4.917)	0.266	10/25 (40.0 %)	2.067 (0.708-6.035)	0.184	0/12 (0.0 %)		
DET (2)									
CS 0-1.0	9/14 (64.3 %)	1.0		7/14 (50.0 %)	1.0		1/9 (11.1 %)	1.0	
CS1.1-1.5	16/26 (61,5 %)	0.889 (0.231-3.425)	0.864	14/26 (53.8 %)	1.167 (0.318-4.284)	0.816	5/16 (31.3 %)	3.636 (0.353-37.457)	0.278
CS 1.6-2	15/20 (75.0 %)	1.667 (0.376-7.394)	0.502	13/20 (65.0 %)	1.857 (0.461-7.482)	0.384	6/15 (40.0 %)	5.333 (0.523-54.344)	0.158

Supplementary Table 2C: Clinical outcomes based on cohort score and number of embryos transferred in female patients ≥ 35 after the transfer of GQE.

SET (2)	CPR	OR (95CI)	P	LBR	OR (95CI)	P	MPR	OR (95CI)	P
CS 0-1.0	17/48 (35.4 %)	1.0		11/48 (22.9 %)			0/17 (0.0 %)		
CS 1.1-1.5	9/22 (40.9 %)	1.262 (0.448-3.556)	0.659	7/22 (31.8 %)	1.570 (0.511-4.818)	0.431	0/9 (0.0 %)		
CS 1.6-2	8/17 (47.1 %)	1.621	0.398	6/17 (35.3 %)	1.835	0.322	0/8 (0.0 %)		

		(0.528-4.973)			(0.552-6.098)				
DET (2)									
CS 0-1.0	7/20 (35.0 %)	1.0		6/20 (30.0 %)	1.0		1/7 (14.3 %)	1.0	
CS 1.1-1.5	22/38 (57.9 %)	2.554 (0.831-7.842)	0.102	18/38 (47.4 %)	2.100 (0.666-6.625)	0.206	5/22 (22.7 %)	1.765 (0.170-18.321)	0.634
CS 1.6-2	14/25 (56.0 %)	2.364 (0.704-7.939)	0.164	13/25 (52.0 %)	2.528 (0.734-8.709)	0.142	3/14 (21.4 %)	1.636 (0.138-19.387)	0.696

Data are shown as ratio (percentage) and were tested with a generalized linear model (binary logistic).
Embryo quality 2 = good quality embryo (GQE or GQE/GQE), CS = Embryo cohort score, CPR = clinical pregnancy rate, DET = double embryo transfer, LBR = live birth rate, MPR = multiple pregnancy rate, OR (95CI) = odds ratio (95 % confidence interval), P = p-value, SET = single embryo transfer.
